# Building Care in the Heart of the Moment – Exploring the Process of Collaboration for the Making of Flexible Assertive Community Treatment

**DOI:** 10.5334/ijic.9832

**Published:** 2026-06-22

**Authors:** Ulrika Bejerholm, Daniel Lindqvist, Sofie Westling, Elisabeth Argentzell, Annika Lexén, Marcus Knutagård

**Affiliations:** 1 Department of Health Sciences, Mental Health, Activity and Participation Lund University, Lund, Sweden; 2 ReLife Centre, Lund University, Lund, Sweden; 3 Department of Psychiatry, Skåne University Hospital, Lund, Sweden; 4 Department of Clinical Sciences, Unit for Biological and Precision Psychiatry, Lund University, Lund, Sweden; 5 Department of Clinical Sciences Malmö, Psychiatry, Lund University, Malmö, Sweden; 6 Department of Urban Studies, Malmö University, Malmö, Sweden

**Keywords:** integrated care, person-centered, recovery, mental illness

## Abstract

**Introduction:**

Professional and organisational collaboration of integrated services, like Flexible Assertive Community Treatment (FACT), plays a pivotal role in ensuring continuous and coordinated care for persons with complex mental health needs. While some research reflect team members’ view of FACT or program fidelity, no research has explored the social practice of FACT and how collaboration evolve and sustain.

**Aim:**

The aim is to explore the process of collaboration for the making of the integrated service of FACT.

**Methods:**

A constructivist grounded theory design helped to collect and analyse empirical evidence of 28 interviews with multiprofessional FACT team members and managers, in 2021 (n = 14) and 2022/2023 (n = 14) in Sweden.

**Results:**

*Building care in the heart of the moment* was constructed as the core category reflecting the timely process of FACT, while the sub-categories of 1) *Embracing empathy and holistic view with users’ best interest at heart*, 2) *Openness, curiosity and genuine interest for each other*, 3) *Borders across disciplines and services get erased yet sharpened*, and 4) *Orchestration of the team and leadership underpins sustainable collaboration* reflected critical ingredients and passages of collaboration.

**Discussion:**

Sustainable collaboration required organisations to support team members’ transition from individual or entrapped social niches into an enabling niche, supportive team environment. Cultivating a social learning space may reduce uncertainty and enables timely, skillful action through which relationships, expertise, and performance develop.

## Introduction

Contemporary mental health services (MHS) require integrated services and professional and organisational collaboration to meet recovery goals of service users with complex mental health needs [1,2,3]. Modern care policy emphasises person-centeredness [4,5], and integrated care models attending to both acute crisis and long-term needs. Continuous and coordinated care and support are considered cornerstones in such high-quality support [6]. However, current MHSs remain fragmented, involving diverse interventions such as medication, single and time-limited interventions, e.g., crisis management [7,8], psychotherapy, inpatient and outpatient care, social and community housing support [9,10,11]. Services typically have clear dividing lines between their policy and organisation and fail to provide integrated care for those in most need [8,9]. In response to this, managers in county council of Region Skåne, municipalities, service users, user representatives, and researchers decided to implement the FACT-model, developed in the Netherlands [12]. The intensity of care is ‘upscaled and downscaled’ by a multidisciplinary team to fit user needs at critical points, to foster a seamless transition in which participant-professional relationships are maintained throughout chain of events. FACT has been implemented and studied in several countries. Single cohort prospective studies [13,14,15,16] and a quasi-experimental controlled study [17] indicate results of fewer inpatient days, admissions and outpatient contacts, while qualitative research found shared caseload to improve work environment, crisis interventions, and quality of care [18]. FACT is feasible to implement [19,20,21], with some program drift in larger geographic or rural areas [20]. Common to these studies is the close connection to fidelity, i.e., how delivery aligns with manual-based assessments and monitoring [22]. While this quantification and objectivation of practice help to differentiate services and results, it does not promote knowledge about social processes and formations related to experiences of the team environment [23]. Furthermore, preparatory and ongoing actions to mitigate collaborative boundary problems across team members and services are neither studied, yet essential for sustainable implementation [1]. It is therefore critical that the expertise taught and monitored through fidelity assessments is untangled from the situated social practice of FACT. Specifically, it is necessary to understand how collaboration processes foster an enabling team environment that benefits users. In this sense, the mere existence of FACT procedures does not ensure their use.

Since collaboration is by nature relational, inspiration is drawn from strength and social niche theory. The strength perspective stresses that even in the midst of suffering there are strengths and potentials, and that problems can be raised above [24,25]. This contrasts with diagnosis-, deficits-, and pathology-focused approaches common in traditional MHS [2,5]. A strong environment emerges when professionals are connected to both their individual but also collaborative strengths [experience, knowledge, skills, sensitivity] [26]. A social niche is formed through transactions between individuals and their environment, shaping the environment in which interactions occur. Taylor [27] views the enabling niche as the environmental analogue of individual strength. Sullivan [28] argues that enabling niches provide opportunities and experiences that facilitate growth and achievements. Such niches foster absence of stigma and enable social connections and community [26]. In contrast, individuals in entrapped niches struggle to engage beyond their own. In education management theory [29], collaboration competence is defined as a group’s ability to interact and achieve outcomes exceeding individual contributions. This concept involves a shift from individual competencies to relational networks that make complex organisations possible. From these perspectives, MHS can be understood as entrapped niches, shaped by medical models, individualistic perspectives, and fragmented services that limit the development of social environments. Collaborative practice may therefore be a key mechanism for implementing recovery-oriented and person-centered services such as FACT. However, the collaboration process underlying successful implementation remains underexplored.

To our knowledge, no research has explored the collaboration processes through which competencies developed when professionals from different disciplines and services work together in a FACT team. The aim is to explore the collaboration process for the making of the integrated service of FACT. Specifically, the study seeks to understand critical prerequisites for collaboration, focusing on social processes that help untangle demarcation problems among team members and foster enabling niches that support service users’ mental health and recovery.

## Methods

Different ontological perspectives underlie our work. Objectivism forms one, since phenomena have been encountered beyond our own intentions, as the team emanates from the FACT model in daily practice. Yet, objectivism stands in contrast to the construction of the team and how team members collaborate and create care through co-production with users over time. The understanding is based on team members’ and managers’ descriptions of their experienced reality of collaborating in service delivery. Thus, it is not a copy of reality as in objectivism, but social processes and situations are interpreted by them. Therefore, hermeneutic epistemology and qualitative interpretative tradition are adopted through a circular movement between the whole and its parts, rather than testing and falsifying an idea. Furthermore, it is argued that a process-oriented methodology is, compared to survey data or manifest content analysis, better suited to reveal dynamics and social processes relevant to user distress. Thus, a constructivist grounded theory was chosen to guide sampling and analysis of empirical data of team members’ and managers’ collaboration process, stretching over a period of 2.5 years [30]. Researchers use their pre-understandings and multiple viewpoints from MHSs (EA, UB, AL, DL, SW), social work (MK), precision medicine (DL), recovery-oriented services (e.g., FACT, Peer Support, Individual Placement and Support, Brief Admission) (EA, UB, MK, AL, SW), implementation research (EA, UB, AL, MK), and constructivist grounded theory research (EA, UB, AL). This means that a theoretical lens is applied both in constructing theory from empirical data and in framing initial interview themes [31]. Although grounded theory is inductive and that theory is grounded in the material, it is increasingly argued that grounded theory also involves deductive reasoning, as researchers’ pre-understandings and theoretical lenses are present in analyses, as in the present study. Furthermore, interviewees were gradually included in accordance with theoretical sampling used in constructive grounded theory [30].

### Setting and participants

The research setting was purposefully selected to include team members and managers of two FACT-teams involved in the research project called *Ups & Downs in Mental Health* (UDiM-FACT trial) with the overall aim being to improve our understanding of whether FACT can significantly affect clinical and personal recovery among persons with complex mental health needs, e.g., mood-, personality-, substance abuse-, attention-, and/or post-traumatic stress disorder, with high rates of comorbidity (FORTE Dnr 2019-00073; ISRCTN Registry, ISRCTN11268588). The outpatient teams are situated in the county council of Region Skåne, in two medium-sized cities with a catchment area of 11 municipalities. In the 1^st^ round, the following team members and managers (n = 14) were individually interviewed; psychiatrist (n = 1); nurse (n = 1); peer support worker (n = 1); social workers (counselor, coordinator, housing support) (n = 3); psychologists (n = 2); occupational therapist (n = 1); Individual Placement and Support employment specialist (n = 1); addiction specialist (n = 1); process-, team-, and municipality managers (n = 3). In the 2^nd^ round, nurses (n = 3), assistant nurse (n = 1), social workers (coordinators, housing support, aid officer) (n = 8), team leader (n = 1), and medical secretary/receptionist (n = 1) were interviewed. They were gradually included according to purposeful sampling of FACT team representation, and then theoretical sampling (e.g., change of attitudes, behaviour and disciplinary roles, critical role models and “glue” for collaboration, collaboration affecting user health, and required capacity and resources). Since the 2^nd^ round occurred once teams had stabilised, continued theoretical sampling focused on outreach- and organisation-related challenges within municipalities, and sustainability.

### FACT intervention

FACT focuses on health where impairments are not solely ascribed to users’ attributes and capacities. Rather, environmental conditions at structural, organisational, and interactional levels form a supportive and enabling environment, bridging obstacles between fragmented care, inaccessible community environments, situational demands, and individual capacities [32]. According to WHO, such person-centred and integrated health services mean placing users’ needs, not diseases, at the centre to promote user participation. Such care emphasises relationships and users’ own value, preferences, resources, and strengths [33]. FACT is a recovery-oriented intervention [2,22] and was developed in the early 2000s by Dutch mental health professionals to address the inflexibility of the Assertive Community Treatment [ACT] model [12], focusing on intensive outreach for persons with psychosis at risk of hospitalisation, homelessness, and neglect [34,35]. FACT accommodates fluctuating support for a broader range of needs in vulnerable populations [12,35]. When a user’s condition worsens, the FACT team ‘upscales’ to ACT-level support, while during periods of stability the support is ‘downscaled’ to a less intensive level. The multidisciplinary team comprises psychiatrists, nurses, psychologists, social workers, employment specialists, and peer support workers. In the Swedish version [36], occupational therapists are included, differentiating it from the original model [37]. FACT supports participants in their own environments regarding their daily activities, housing, and social roles, promoting recovery [20].

### Data collection

Data collection was performed during two periods Oct 2020–May 2021 (1^st^ round) and Oct 2022–April 2023 (2^nd^ round). An interview guide with open-ended questions was developed, starting with, “Can you tell us about your experience of collaborating with others in the team?”. In the 1^st^ round, team members and managers were interviewed by a research assistant and authors MK and AL, and in the 2^nd^ round by MK. Interviews lasted 45–60 minutes, were audio-recorded, and transcribed verbatim. No personal data was collected, and interviewers had no previous contact with interviewees. All received information about the study and provided informed consent. The study is part of a process evaluation of UDiM-FACT trial, with ethical approval (Dnr 2019-02866), conducted in accordance with the EU General Data Protection Regulation.

### Data analysis

The analysis process began with the first interviews, in agreement with theoretical sampling principles, with data collection and analysis proceeding concurrently to inform the interview guide and theoretical sampling. The research assistant (main interviewer) conducted initial line-by-line coding using a constructivist approach that emphasised participants’ meanings and actions. Early analytic memos were written after each interview to capture emerging ideas, questions, and reflexive considerations. The research assistant and EA, UB, MK and AL met after each interview during the 1^st^ round to discuss early memos, emerging codes, and preliminary categories. These discussions supported constant comparison, critical reflection during interpretation of the material, and informed iterations that helped to refine or add interview questions and greater focus on emerging concepts. For example, early discussions revealed that broad questions resulted in general answers, prompting more detailed questions about colleagues and changes in participants’ attitudes over time. Meetings also functioned as analytic and reflexive sessions, in which all researchers discussed pre-understandings and theoretical underpinnings and how these might influence emerging findings and co-construction of meaning. The tentative analysis process was facilitated by UB and MK, while analytic rigor was strengthened through ongoing team discussions. Microsoft Word was used to facilitate data analysis. Separate documents were used to organise line-by-line, focused, and axial coding, memo-writing, and categorisation, with codes and memos collapsed through iterative comparison. The analysis process culminated in the identification of a core category and related categories [30].

Credibility was enhanced by interviewing all multiprofessional team members and theoretical sampling, including the 2^nd^ round of interviews that covered newly employed staff and organisational changes within municipalities, addressing sustainability and theoretical saturation [38]. The novel understanding of the FACT collaboration process addresses originality and resonance and, together with its usefulness for providing new insights and research directions, underscores trustworthiness regarding depth and richness [30,38].

## Results

*Building care in the heart of the moment* is constructed as the core category, reflecting the overall creativity and dedication to meeting service users’ mental health needs when and where needed, in the moment.

“Being there, the speed, at the right moment, is key for the target group. We are all around and just kind of do it”

(Manager, Social Services, 1^st^ round)

Team members experienced the collaborative process across disciplinary and service boarders as mitigating hopelessness, compulsory care, threat, violence, and suicide attempts in relation to the target group. Managers addressed collaboration to alter care use statistics, possibly with a decrease emergency admission and compulsory care and an increase of opportunities for health and recovery.

“A user, not approachable, has not had any compulsory or inpatient care for two years. Now the participant is studying at the university, living alone and having a pet. It is a consequence of collaboration (…) We work together in whatever situation participants finds themselves in”.

(Social Worker, Housing Support, 2^nd^ round)

**Figure 1:**
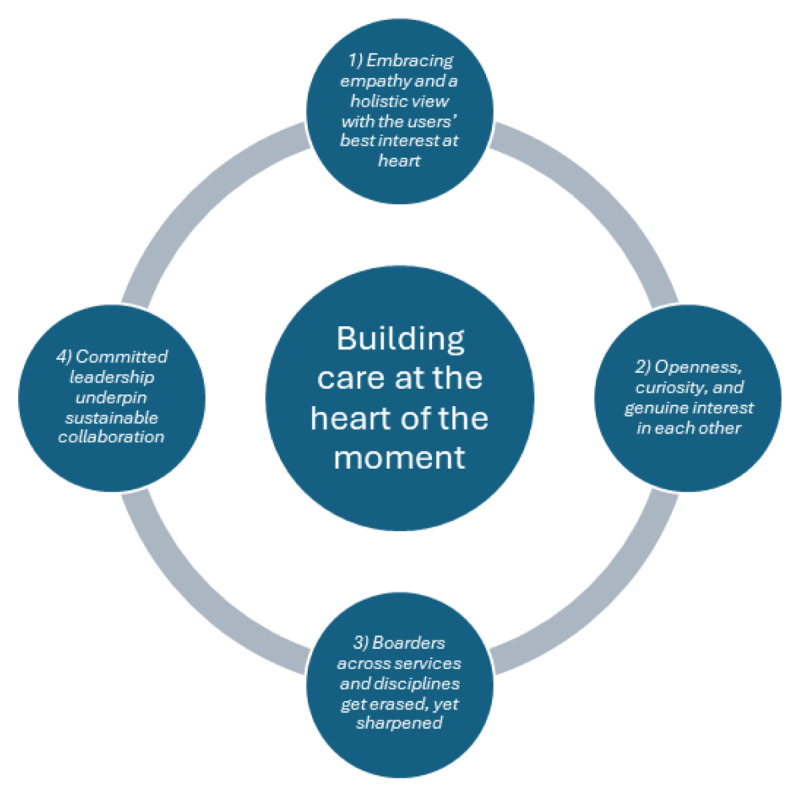
Core category and supplementary categories of the collaboration process of FACT.

Four categories reflect critical passages or elements of the collaboration as a craft and process that together explain how *Building care at the heart of the moment* come across to users (Figure 1). *Embracing empathy and a holistic view with users’ best interest at heart* reflect the common belief, vision and mindset held by team members and managers from the very start. *Openness, curiosity, and genuine interest in each other* are components needed to build a psychological safe environment that enables collaboration around user goals, allowing *Boarders across services and disciplines get erased, yet sharpened* to balance learning and performance. Further, *Orchestration of the team and committed leadership underpinned sustainable collaboration* across initial, intermediate, and long-term phases. Together, the categories depict essential elements for developing collaborative competence and fostering enabling niches and social learning. In the results, the constructed tentative theory is further elaborated.

### Embracing empathy and a holistic view with users’ best interest at heart

Team members described shared compassion, empathy, and had corresponding values and interests for the user group from the onset. This category reflects this critical component enabling early collaboration processes, learnings and team formations. Although several team members explained having no previous experience of integrated services, such as FACT, their best interest for the target group provided a platform for building collaboration and working together to find solutions that matched user needs.

“Everything is new, collaboration does not work by itself. But it’s simple here, to help the patient, to work person-centred”

(Social Worker, Coordinator, 1st round)

Although team members employed a holistic view and acted in service users’ best interests, some users were perceived as being reluctant to trust them. This reluctance was described as partly attributed to service users’ history of compulsory care and repeated treatments with little progress, and partly to their perceptions of professionals being distant. In such circumstances, the peer support team member could more easily gain trust and enable user engagement. The experienced knowledge of peer support further contributed to a language and work culture of embracing empathy, as well as to new types of questions and encounters with users.

“It was easier for the peer supporter to gain trust and contact at first (…) It can certainly feel difficult that professionals within the municipality and psychiatry don’t usually open-up. The professional facade. What probably happens in FACT is that you go in and out of having a more personal role and change what you are and how to relate.”

(Social Worker, Coordinator, 1st round)

“Yes, out of empathy, and out of the tremendous human love and the absolute belief that this is how people rise from the most horrific experiences. And you have seen so many land on their feet again. To be able to sort of think that with everyone we meet, I don’t know which of them won’t stand up. You can’t assume that any of them wouldn’t.”

(Peer Supporter, 1^st^ round)

However, it was when relationships between the users and team members became real and meaningful that good intentions benefited users. Team members described the importance of “walking by their side”, being real and personal in the encounter, and building relationships over time. Embracing a holistic view became natural, as collaboration was directed toward a shared communicated user goal. Team members interpreted their united mission as an umbrella that shelters users, keeping them safe and stable while also enabling their recovery journey. Team members articulated their emphatic and holistic view in tandem with their perception of traditional MHSs.

“No one has succeeded despite treatment homes and round-the-clock care since adolescence. We now try Brief Admission, support work, and collaborate with housing staff. Completely different quality of life because of FACT. Someone has taken the overall approach where the relationship means a lot.”

(Nurse, 2^nd^ round)

“We don’t have much focus on diagnoses and take people as they are. We don’t have many suicides; we manage to catch them.”

(Social Worker, 2nd round)

Embracing empathy and a holistic view facilitated a shared understanding among team members and was associated with a gradual scaling down of emergency and inpatient care. Team members contrasted this approach with experiences in traditional settings, described as involving multiple, reactive, diagnosis-driven interventions and limited coordination. In such settings, professional roles and organisational structures were perceived to shape practice more strongly than the empathic and holistic approach in FACT. One team member reflected on how collaboration enabled this.

“Remember that the municipality is an authority, not breaking the law. So, we need to think outside the box and be flexible and fearless. Need to see the benefits of this and not get hung up on what doesn’t work”

(Social Worker, Coordinator, 2nd round).

### Openness, curiosity, and genuine interest in each other

This category represents qualities needed to support collaboration around user goals. These qualities “or glue” are present throughout collaboration but were most prominent during instrumental learnings of FACT, and at times when the caseload was lower.

“We are pretty open here, that we can say what is on our minds and even though maybe not everyone agrees, it is actually completely okay to say things, and we can have those discussions.”

(Addiction specialist, 1^st^ round)

“Yes, our different strengths in the team, you are not best at everything, but we have different strengths and try to take advantage of those.”

(Psychiatrists, 1^st^ round)

“All professionals get enriched of each other’s knowledge and practice, also as persons.”

(Psychologist, 1^st^ round)

Lack of prestige was described a social norm that rubbed off on new team members. It was a personal and professional quality that promoted a good tone, creating a social environment in which members feel safe and dare to take up space, even when they feel vulnerable and make mistakes. It was evident in the material that it became possible to present oneself, share ideas, and discuss, debate and learn from each other. In turn, such an open social climate was perceived to promote trust, confidence, self-awareness, receptiveness, responsiveness, a sense of humour, fearlessness, innovation, creativity, and adaptability.

Although team members valued each other and were curious, interest in specific roles and knowledge varied with the characteristics of the caseload. For example, newly recruited users often required acute and ongoing assertive outreach treatment to stay alive, secure housing, or avoid compulsory care. Accordingly, interest in roles and knowledge related to psychosocial interventions and personal recovery increased once users entered more stable phases. Thus, a sense of belonging and identification with the team may vary in intensity and be temporary, particularly early on.

“Characteristics like commitment, spark, responsive, persistent, patience and liking the target group (…) and try to develop team spirit”

(Assistant Nurse, 2nd round)

“Open climate, I get lot of support. You get different input with different disciplines and personalities. A kind of support that does not get stuck. There we can lift anything. Very nice climate. Never insecure. Open. Everyone loves being with us. Not prestigious. Everyone has qualities and experiences that make it better. Let each other go and talk about who is best suited. Very comfortable in the team.”

(Social Worker, Housing Support, 2nd round)

### Borders across disciplines and services get erased, yet sharpened

This category reflects that balanced competencies were experienced to land promptly “in the lap of users”. In developing collaboration, it was evident that no single discipline within the team or service (county council or municipality) is claimed to be superior to another. Initial collaboration actions were built from various experiences and perspectives within the multiprofessional team, all members keeping an open mind.

“No profession creates better contact with patients. But it’s about building a relationship what works.”

(Social Worker, 1^st^ round)

“At previous outpatient unit, where I then was senior psychiatrist, I decided, like doctors usually do (…) Here, I was quite open and did not need to know everything in advance.”

(Psychiatrist, 1^st^ round)

Previous disciplinary borders were challenged. Disciplinary roles that were less visible and powerful within county council MHSs (e.g., peer supporter, assistant nurses, occupational therapists, and social workers) became noticed and valued and believed as entailing rich knowledge about health and meaningful ways to connect with users. For example, a peer supporter can collaborate on equal terms with a psychiatrist and help facilitate relationships and treatments through open dialogue with users in their homes.

“I feel safe and invited. A psychiatrist has kind of the same power, not more or less than me.”

(Peer Supporter, 1^st^ round)

“We work as equals, taking advantage of each other’s skills. No hierarchy.”

(Nurse, 2^nd^ round)

Previous experiences of the county council are highlighted to contrast the collaborative environment enabling niches in FACT. In these services, diagnosis, symptoms and social problems are described as decisive for what discipline is viewed as superior, with little attention to user needs. At first, team members from the municipality may feel less important than medically trained team members. In this sense it is critical to let go of borders:

“Not always so easy to have had the overall grasp and relationship with users in the municipality and then leave it to someone else in the team. Not easy. Before, there was this personal feeling of inferiority compared to others. But you need to sit down and think through what is important, based on all experience with the user. So better now.”

(Social Worker, Housing Support, 2^nd^ round)

Furthermore, contradictory assignments and areas of responsibility between the county council and the municipality have been experienced as fostering a lack of collaboration between these services. A process leader explained “how it was before”:

“When a user gets ill again: ‘This is not our responsibility, it is the county council’s’ (…). Then a homeless person could be sent around, also between municipalities, blaming each other and believing that others did a bad job.”

(Process manager, 1^st^ round)

Yet the organisational boundaries were described to weaken when collaboration became more relational and extended beyond formal responsibilities. Over time, collaboration across services is stated as unique and experienced as having no wall between. It is described as daily innovations to counteract that users are “sent around”.

“We are now crossing the boarders.”

(Social worker, Housing Support, 2^nd^ round)

“In the past, (…) the police and ambulance were called. We believed that the county council would do this or that. Opinions, which they also had about us. We never met. Now we have started talking (…) listening and helping each other. Easier to work with seamless support that we can carry out and get hold of all professions needed. Quick responses. Terribly difficult situations, we back each other up. The team has different skills, and we use that and each other.

(Social Worker, 2^nd^ round).

The removal of borders required a process of learning collaboration, a social practice where innovation and trial-and-error became central, a space where team members interact and learn. It concerned the action of building care together, over-and-over, as team members moved away from their own enclosed expert role or individual niche towards being equally valued and critical to each other and for the user. Learning collaboration was a sharp and transparent balanced act between accepting and identifying with an emerging discussion during user-oriented team meetings and voicing diverging ideas and learning from mistakes. This passage involved expressed uncertainties of one’s own and others’ matters. During encounters with users, the balancing act concerned prompting oneself when needed. Being alert and prepared to that “everything can change in 5 minutes”, depending on the moment at hand. Advancing and retreating in a careful, tactful, and responsive manner with others were described as key dynamics. However, the balance act of timing and pacing required self-awareness and ongoing reflection on one’s own and others’ perspectives and disciplinary roles, always for the benefit of service users, like a symmetry between voice and silence.

“Professionals have many similarities and differences, facilitate themselves differently in different situations. The pacing varies, for everyone.”

(Psychologist, 1^st^ round)

“You need to make your own decisions and keep a cool head, to consult each other but be confident in your own role.”

(Assistant Nurse, 2^nd^ round)

“We are team players and it is about the team’s patients, not like in regular services where it is “my patients.”

(Medical Secretary, 2^nd^ round)

Team members developed intuition of how to pace themselves and position one another in emergency situations and when distributing cases. Given the competencies required, team members suggested that newly graduated professionals may struggle with this delicate balance act, as they often lack experience and confidence in their disciplinary role.

A persistent collaboration boundary was identified at the interface between the FACT team and emergency or inpatient MHSs. Team members described situations in which their assessments of inpatient needs were not always recognised, and users were being sent home. Brief Admission with self-referral contracts was described as providing a structured pathway between FACT and inpatient services, which was analytically understood as reducing this remaining border.

### Orchestration of the team and leadership underpins sustainable collaboration

This category reflects organisational- and team-level components that were critical for sustaining collaboration over the 2-year interview period.

“Collaboration demands orchestration, planning, and logistics.”

(IPS Employment Specialist, 1^st^ round)

To enable common grounds for team members to collaborate, the orchestration of the team requires both vertical and horisontal structures, organisation, and leadership. At the vertical level, it was considered essential that FACT corroborates current national and regional policies on integrated- and person-centred services. However, preparation and planning at the local organisational level also emerged as important before forming a team. Since organising collaboration across service and disciplinary boundaries was experienced as a novelty, actions were taken to engage future partners in managerial positions to discuss needs, resources, decisions and strategies. In this early phase, MHS and process managers recounted how they reached out to and visited each municipality to address legitimacy and acceptance of FACT. Across accounts, the need for such efforts was emphasised, since integrated services for this target group are entirely lacking, and users are believed to fall between the cracks. It was further noted that no additional financial resources were needed, but a mandatory collaboration agreement was developed, written, and signed between county council and municipality managers. This contract provided a clear local policy for integrating services and collaboration. Likewise, a contract was also developed for users to agree/disagree to that professionals share information across organisational borders. Both policies and contracts extended beyond the operational FACT team and were portrayed as having a ripple effect of “a pebble in the pond”, with trickle-down effect across municipalities. Furthermore, managers emerged as key players for forming steering- and working groups, and other incentives, to organise collaboration across services and team members. In this orchestration, managers own attitudes and views were highlighted as important to underpin such efforts.

“Leaders need to take ownership of the matter on a structural level to build a frame in which a collaboration process and network may be built.”

(Manager in Municipality, 1^st^ round)

“The agreement is important for collaboration. There is a line in the organisation, so it doesn’t fall into oblivion. FACT has led to change of how work is done in social services, with now more mandate to act. You need to be clear with (operational) managers about what is needed, rights to document and the like. Trust-based leadership from managers is important. If managers don’t agree, there is a need to explain the consequences for social work, involvement of much more staff, and clients being worse off, etcetera.”

(Social Worker, Coordinator, 1^st^ round)

The horisontal organisation of integrating services involved collaboration among several municipalities, grasping a larger geographical area. It was essential that coordinators (social workers) functioned as ambassadors in each municipality to address issues and build networks locally. This was experienced as an important yet challenging process without extended resources and existing organisational structures, responsibility areas and belief systems. To facilitate a collaborative culture, coordinating team members meet monthly to develop shared understandings and strategies. At the team level, the orchestration of collaboration is initiated and structurally sustained through the FACT model (see Intervention). It provided “hardware” guidance of “what” to do” (e.g. education, daily meetings, team building, mirror sessions, supervision, and team meetings).

“In the beginning, there was a lot of focus on the forms of collaboration, the frameworks and the structure and so on. Everyone was stressed and we had timetables to keep. It has been a process.”

(Occupational Therapist, 1^st^ round)

Yet the “software” or guidance of “how” to enable, perform and sustain collaboration, was reflected in accounts of leadership quality. It was evident that the ideal leadership style of team managers is trust-based, and analytically linked to team psychological safety, enabling an open climate, communication and disciplined experimentation. Leaders were described as listening, managing and structuring meetings, being present and candid, and engaging in the moment to actively promote a democratic social space where everyone has a say. When the team is operative, leaders were depicted as mitigating rigidity and frictions as needed, framing discussions and putting performance, solutions, and failure into perspective. Leaders were described as fearless, facilitating performance, and integrating diverse competencies. Leaders were further seen as hiring and involving new team members in open dialogue, contributing to the initial social learning phase of collaboration.

“We are a flat organisation. Team manager is central for dialogue and climate and collaboration, captures what we really think. All input is equally valuable (…) Staff has been replaced, but it remains stable (…), management is stable over time.”

(Social Worker, Housing Support, 2^nd^ round)

Staff turnover of coordinating team members and managers within the municipality was experienced as disrupting collaboration. When so, the team reflected on the impact on collaboration and how to mend the link. In the most rural parts, team members eagerly mended knowledge gaps or temporarily replaced one another to maintain the social link of collaboration. Sustainable collaboration also hinges on the fact that municipalities operate and function differently.

“It applies to, keeping it alive, and it is different in different municipalities, very chaotic. Works well in municipalities where tasks can be delegated and there is simpler organisation.”

(Social Worker, Coordinator, 2^nd^ round)

“Municipalities, all municipalities, function differently (…) This challenges collaboration. So, members in the team must constantly update how they work and how they can provide support. It affects FACT indirectly.”

(Nurse, 2^nd^ round)

Finally, since an enabling environment had been built and the collaboration process was sustained, new staff were perceived as taking on their role quite easily. In such situations, turnover was at times reframed from a challenge into a potential, as new team members were seen to bring new perspectives, innovations and additional qualities to the team.

“FACT has become part of the backbone after two years of practice when the organisation has settled.”

(Social Worker, Coordinator, 2^nd^ round)

## Discussion

This study confirms established principles of collaboration and extends them within the FACT context. It advances understanding of FACT collaboration processes and the social prerequisites for developing enabling niches to support service users’ mental health and recovery. The results align with literature on interprofessional collaboration in integrated care [39], framing collaboration as ongoing behavioural and relational work [39]. Building on the concepts of entrapped and enabling niches, the study has clinical implications, as they may help managers and team members discern whether the team’s social environment supports collaboration processes. In line with the WHO [32], mental health and impairments cannot solely be ascribed to users’ own attributes and capacities. Rather, an enabling niche of the team environment helps bridge obstacles arising from fragmented and non-adaptive care, inaccessible environments, situational demands, and individual capacities, aligning with the core category, *Building care in the heart of the moment*. Through the enabling niche of the team, service users’ suffering may be eased, at the right time and place. Qualitative studies on user experiences of FACT corroborate this stand, showing that service users themselves become team members [41] and that genuine relationships support mental health, participation and personal recovery [42]. Taken together, the findings not only resonate with existing literature, but also point toward distinct processes through which collaboration is enacted and sustained.

Pertaining to the idea of enabling niches, the notion of social learning spaces [43] reflects the analytic patterns of the results and their importance for collaboration. The Wenger-Trayner theory is used to illuminate how team members generate a social learning space in which learning is shared, encouraged and cultivated, as in the present study. The first two categories (see Figure 1) are particularly resonant with the key dimensions: 1) Caring to make a difference: Participants engage because they see an opportunity to enhance their ability to affect the world or themselves in meaningful ways; 2) Engaging uncertainty to making a difference, exploring what it means, and finding ways to achieve it; 3) Paying attention: Actively listening and responding to feedback, leading to new understandings, insights, and adjustments. The first category “*Embracing empathy”* is reflecting a shared commitment to values, vision, attitudes and governance that together foster a space for social learning. While these dimensions align with existing literature, the present study specifies how they are enacted within FACT, particularly through its integrated and time-sensitive mode of collaboration. The first category constitutes a prerequisite for the second category, “*Openness and curiosity”*, capturing relational qualities that function as the social glue of collaboration needed to develop an enabling niche [25].

The results may further be interpreted in light of psychological safety [40]. The core category, *Building care in the heart of the moment*, reflects the temporality of immediate experience, in which relationships, expertise are created, conditions that foster a fearless environment [40], enabling team members to share knowledge and trust each other’s contributions. Psychological safety is especially important for minority groups in a workforce. For example, peer supporters shared knowledge and worked on equal terms with other team members. The findings demonstrate how these conditions enable moment-to-moment collaboration in FACT. Thus, it is not sufficient to recruit highly trained professionals if collaboration fails at critical points in time and place in response to user needs [40]. When confined to individual niches, professionals may hesitate, fearing doing wrong and risking relationships. Therefore, collaboration should entail a prestigeless, dynamic, and innovative teamwork to enable sharp user-centres decisions, as reflected by the third category, “*Boarders across disciplines”*. Thus, borders across team members and services must fade to provide precise team intervention, as reflected by the core category. It is thus critical to support collaboration by moving from entrapped or individual niches toward enabling niches, particularly at the county-municipal interface. According to Sennett [44], daily innovation and experimentation demand skillful execution which needs to be developed and honed like any craft, grounded active listening dialogue rather than confrontational debates. True collaboration requires ongoing learning, problem-solving, and imagination, a craft captured by the two final categories where team members engaged in constant balancing and pacing act in relation to colleagues and user needs. Managers were described as “fearless” and was interpreted as contributing to psychological safety and to social learning space in which team members were given a voice from the start.

In contrast to conceptual and framework-based research on collaboration in integrated care, which primarily identify general behavioural conditions for effectiveness, this constructivist grounded theory offers an empirically grounded account of how collaboration is enacted, sustained, and repaired over time in a specific integrated mental health context. Rather than focusing solely behaviours, the analysis foregrounds collaboration as the social practice in FACT, showing how enabling and entrapped niches emerge in everyday encounters, how boundaries between disciplines and services are continually erased and redrawn, and how collaboration is shaped by temporality, uncertainty, and fluctuating care demands. The findings extend existing research by detailing the role of orchestration, formal agreements, and cross-municipality coordination as practical mechanisms in complex and fragmented service systems. By explicating these processes, the study moves beyond identifying what supports collaboration to illuminate how collaborative competence is developed and maintained. While previous FACT research has highlighted the importance of collaboration [20], results provide empirical insight into the social practice and identifies process elements that are critical for team performance and service integration. Deliberately strengthening collaboration processes may mitigate program drift, even when FACT spans large geographical and rural areas [20]. Leadership emerged as essential for planning implementation and addressing vertical and horisontal uncertainties in advance, consistent with previous research [1,45,46,47].

Previous research indicate that FACT promotes the psychosocial working environment, e.g., increasing control, reducing job-strain and enhancing job-satisfaction for team members [18]. Rather than merely confirming earlier findings, the present study extends this knowledge by showing how enabling, rather than entrapped, niches are produced through cross-boundary collaboration across county council–municipality interfaces, multiple municipalities, and rural contexts. These processes actively create and maintain psychosocial working conditions in FACT, helping to explain mechanisms underpinning team sustainability, performance, and quality. Central to the temporality of collaboration, how care is continuously scaled up and down over time and built “in the heart of the moment” in response to shifting user needs.

### Methodological considerations

The pre-understanding of the researchers is viewed as both a strength and a limitation. A strength, since researchers reflected the multi-professional composition of the team, enhancing resonance, depth, and richness during theoretical sampling and construction of theory. The consideration of context increased awareness and relevance for clinical practice. Credibility is evident since results revealed novel empirical data and represented team members’ experiences of collaboration, where the theories of social niches, psychological safety, and social learning space resonated with results. Dependability is reflected since the research process was stable over time. The two-year period, however, required that the main interviewer [MC] anchored, coordinated, and facilitated regular reflexive discussions with among researchers (UB, AL). Regarding transferability, the constructed results are limited two FACT context of the study. However, as FACT is a highly manualised and standardised model, the identified collaboration processes and mechanisms may be transferable to other contexts embedded in this specific organisational, normative, and value-based condition. Thus, results support process-level and analytical transferability, rather than broad generalisation.

## Conclusion

The grounded theory helped explore the social practice of FACT. The categories identified key elements of the process while theories on social learning spaces [43] and psychological safety [40] helped to pinpoint, elevate, and broaden the understanding of how enabling niches may be developed. The core category *Building care in the heart of the moment* encapsulates the imaginative and committed approach to addressing service users’ mental health and social needs promptly and effectively. This collaborative climate facilitates the seamless yet nuanced intersection of service and disciplinary boundaries, a delicate act of learning and enacting collaboration. Orchestrated team dynamics and steadfast leadership are essential to sustaining collaboration across initial, developmental, and long-term phases.
